# Supplementary material for: Choosing the right strategies: An analysis of crisis response strategies in Chinese universities
Source: PLoS One. 2025 Apr 8;20(4):e0321777. doi: 10.1371/journal.pone.0321777 (PMC11991675; doi:10.1371/journal.pone.0321777)
Supplement: S1 Table — (DOCX) [file pone.0321777.s001.docx]

**Supporting information**

**S1 Table. Example Sentences for Crisis Response Strategies.**

| **Coding** | **Strategy** | **Example** |
| --- | --- | --- |
| A1 | Attack the accuser | We reserve the right to take further responsibility for the recent online publication of fake news in the name of the university and malicious slander of the university image. (Case No.8) |
| A2 | Denial | During the epidemic prevention and control period, in order to ensure the health and safety of students, also meet the actual needs of students, although the university has formulated and implemented a system of leaving according to the prevention and control regulations of the superior, there is no situation that students are not allowed to enter and leave freely. (Case No.15) |
| A3 | Scapegoat | Last night, our school conducted a regular fall dormitory safety inspection, and there was not a male teacher in a khaki coat involved in the inspection. In response to the unidentified person appearing in the online post, the school has notified the relevant departments and requested assistance in investigation and verification. (Case No.15) |
| B1 | Excuse | During the special period, although the activity was well-intentioned, it was ill-considered and violated the relevant regulations of prohibiting gathering during the epidemic prevention and control period. (Case No.1） |
| B2 | Justification# | / |
| B3 | Transference* | After investigation, it was found that Wang required students to call him father and other behaviors unrelated to teaching and scientific research, as well as improper ways to guide students in the process of study and employment. But it was not found that Wang prevented students from studying a master's degrees in another university and defending his dissertation, occupied students' economic interests, and made students wash clothes and do housework at his home. (Case No.10) |
| B4 | Separation* | After investigation, our graduates have no connection with the people mentioned on the internet. (Case No.16) |
| C1 | Compensation | After the incident, the school attached great importance to it and quickly set up a special working group to communicate with the student's relatives, give spiritual comfort and material care for their lives, and actively deal with the aftermath of the incident. (Case No.7) |
| C2 | Apology | As the college's decision has caused serious harm to Teacher Liu and her family, and has had a bad impact on the society, we express our sincerest apologies here again! (Case No.4) |
| C3 | Commitment* | Thanks to the attention, criticism, and supervision of the school from all walks of life, we will humbly accept, deeply reflect on, and seriously rectify the criticism of the netizens. (Case No.18) |
| C4 | Remedy* | Relevant departments have required the dining units to strengthen the food safety management of the canteen and implement the main responsibility for food safety. (Case No.19) |
| D1 | Investigation* | The school set up a special working group on June 11 to quickly carry out the investigation. (Case No.11) |
| D2 | Information disclosure* | After investigation, it was found that in the process of material review, interview and recruitment of Zhai, the co-tutors, the interview team and Guanghua School of Management had some problems in academic review; Zhai was also confirmed to have committed academic misconduct. (Case No.9) |
| D3 | Explanation* | The activities provided coke and snacks, which were stocked by the resident teachers themselves. The bags were made by the teacher with kraft paper bags, which contained their daily reserve of cookies, coffee and candy, the things in each bag were different, and there were the student's name and birthday wishes on the bags written by the teachers. (Case No.1) |
| D4 | Accountability* | The school decided to stop recruiting post-doctoral students for the co-supervisor, seriously criticized the members of the interview team, and ordered Guanghua School of Management to make a profound self-examination. (Case No.9) |
| D5 | Punishment* | On the afternoon of March 25, the joint meeting of party-government decided to deal with Ni as follows: 1. Ni should immediately apologize to the student and other students in the same research group face to face and make a deep reflection of himself; 2. Inform the entire faculty of his mistakes and criticize him; 3. Stop his teaching work immediately. (Case No.13) |
| E1 | Reminder | In the years of school-running practice, our college has formed a unique art enrollment and selection model and talent cultivation system, cultivated many outstanding professionals in the field of animation for the country, created a number of outstanding works with wide influence, and made important contributions to the development of China's animation industry. (Case No.5) |
| E2 | Ingratiation# | / |
| E3 | Victimage# | / |
| E4 | Endorsement* | On April 26, the Shanghai Market Supervision Administration learned that some netizens reflected the quality problems of Tongji University's meals (salty pork suspected to have parasitic eggs), and immediately organized the relevant regional market supervision bureau to carry out verification and disposal. (Case No.19) |
| E5 | Appeal* | Thanks for the concern and support from the netizen, and hope you respect the rights and interests of our graduates and build a civilized network together. (Case No.16) |
| E6 | Warning* | Here to remind candidates, especially the law talents of the future, to enhance the awareness of rules and integrity, and strictly abide by the relevant provisions of the exam. (Case No.6) |
| F1 | Valued highly* | In the daily monitoring of public opinion on May 23, it was found that several network platforms forwarded the micro blog that "Shaanxi Institute of Foreign Languages of Technology recruits 3 teachers and 2 are the children of internal middle-level leaders". Upon learning of this situation, the school attached great importance to it and immediately set up a special investigation team, which has begun to investigate and verify the problems reflected in the micro blog. (Case No.17) |
| F2 | Concern* | We deeply mourn comrade Wang and express deep sympathy to the families of the victim. (Case No.2) |
| F3 | Condolence* | We are deeply saddened by the loss of such a good comrade and an excellent Party member. (Case No.2) |
| F4 | Gratitude* | We are grateful for the concern from all sectors of society. (Case No.20) |
| F5 | Statement* | Our school has always attached great importance to the construction of teachers' ethics, strictly standardized teachers' behavior, and adopted a "zero tolerance" attitude toward improper behavior. (Case No.3) |
