# Supplementary material for: Choosing the right strategies: An analysis of crisis response strategies in Chinese universities
Source: PLoS One. 2025 Apr 8;20(4):e0321777. doi: 10.1371/journal.pone.0321777 (PMC11991675; doi:10.1371/journal.pone.0321777)
Supplement: Attachment — Submitted filename: Response_to_Reviewers_auresp_4.docx [file pone.0321777.s006.docx]

**Dear Editor and Reviewers:**

We are immensely grateful for the precious time and effort you have dedicated to providing professional and in-depth comments on our article. Based on your invaluable suggestions, we have once again carefully reviewed and revised the article. We believe that after 4 meticulous rounds of revisions, the article has significantly improved. We sincerely hope to gain your recognition and obtain the opportunity for publication.

Thank you once again for the dedication of the editors and reviewers and wish you all the best!

**Regarding revisions required by the editor:**

1.Please review your reference list to ensure that it is complete and correct. If you have cited papers that have been retracted, please include the rationale for doing so in the manuscript text, or remove these references and replace them with relevant current references. Any changes to the reference list should be mentioned in the rebuttal letter that accompanies your revised manuscript. If you need to cite a retracted article, indicate the article’s retracted status in the References list and also include a citation and full reference for the retraction notice.

- Thanks to the editor for the reminder. We re-examined the references individually to confirm that they had not been retracted. Furthermore, as this is a minor revision with limited changes, only one reference was removed during the revision process:

[59]Siomkos GJ, Kurzbard G. The hidden crisis in product harm crisis management. European Journal of Marketing. 1994;28(2),30-41.

**Regarding revisions required by the reviewer #2:**

1. The abstract provides a good summary. State explicitly how university administrators can use these findings in real crisis situations. Provide a sentence on how universities can incorporate these new strategies into their crisis response plans.

- Firstly, we would like to express our sincere gratitude to the reviewer for acknowledging and recognizing our work. The findings of this study can indeed enable university administrators to apply these new strategies in crisis response, which can be incorporated into their crisis response plans. Therefore, based on your suggestion, we have added a sentence in the abstract to convey this meaning. The revised sentence is as follows:

“The findings provided guiding significance for Chinese universities administrators in developing and implementing effective crisis response strategies in real-life crisis situations, enabling them to adjust and optimize their plans by incorporating innovative strategies to enhance the effectiveness of responses.”

- In addition, we want to explain to you that, for your convenience during the review process, we originally intended to highlight the revisions made. However, according to the journal editor's instruction, the main manuscript file should not contain any tracked changes or highlighting, as this will be used in the production process if the manuscript is accepted. Therefore, we will endeavor to explain all the revisions in the response letter instead.

2. The paper justifies excluding third-party narratives, but it does not fully address why stakeholder perceptions were not considered. Since crisis communication is about perception management, future research should examine how students, faculty, and the public perceive these responses.

- Thank you for your comment. The reviewers have provided insightful observations on the core issues of this study. Indeed, we are well aware that the essence of crisis communication goes beyond the unilateral strategic choices of information issuers. Crisis response is a two-way process of perception management, which hinges on whether stakeholders (students, faculty, the public, etc.) truly receive, understand, and accept these strategies. There may be slight deviations between official strategies and stakeholders' perceptions. More importantly, as we have stated in the article, due to the fact that stakeholders' perceptions may be influenced by their own interests, as well as potential emotional factors and biases towards the organization, it is not conducive to maintaining the objectivity of this study.

However, the exclusion of third-party narratives and even stakeholder perspectives in this study is not in opposition to the reviewers' viewpoints, rather, it reflects a different emphasis in research focus. This study focuses on the coding of institutionalized strategies in universities' official responses, and reveals what response strategies Chinese universities choose under the institutional logic of organizational legitimacy and policy compliance when responding to crises. This methodological design does not neglect stakeholders' perceptions but rather delineated them as distinct phases, firstly, we focused on analyzing which strategies the university as the responding entity has employed in the official documents. In the future, based on your suggestions, the stakeholders’ perceptions will be further taken into consideration, representing another independent analytical layer that requires different methodological approaches.

The strategies identified in this study initially demonstrate the strategies applied by universities as response entities in their crisis responses. Official documents, as authoritative sources of institutional intent, enable us to track how universities make strategic choices within recognized discourse frameworks in response to crises. By focusing on universities' official institutional response strategies, it is crucial for identifying systematic patterns rather than individual cases. The research findings can serve as a baseline framework, against which future related studies can measure the degree of alignment or inconsistency in cognitive outcomes. This study does not deny the importance of perception management but rather lays the foundation for the future research.

According to your comments, we have made two changes to the article, one is ROW251-261 about the description of excluding third-party narratives and stakeholder perceptions, and the other is ROW609-616 about future research direction at the end of the article.

“As the purpose of this research is to determine how the Chinese universities communicated about crises in their external communications, news articles or interviews of school staff were excluded to avoid the subjectivity of a third party in the reporting and framing of the universities’ responses. All documentary evidence is officially released by universities. Meanwhile, due to stakeholders perceptions may be influenced by their own interests, and potential emotional factors and biases towards the organization, in order to focus on the coding of institutionalized strategies in universities' official responses, and reveal what response strategies Chinese universities choose under the institutional logic of organizational legitimacy and policy compliance when responding to crises, while maintaining the objectivity of the research, the influence of stakeholders perceptions are also excluded in this study.”

“Furthermore, the strategies identified in this study initially demonstrate the strategies applied by universities as response entities in their crisis responses. However, the essence of crisis communication goes beyond the unilateral strategic choices of information issuers. Crisis response is a two-way process of perception management, which hinges on whether stakeholders (students, faculty, the public, etc.) truly receive, understand, and accept these strategies. In the future, the stakeholders’ perceptions will be further taken into consideration, the findings of this research can serve as a baseline framework, against which future related studies can measure the degree of alignment or inconsistency in cognitive outcomes.”

3. The newly identified strategies are mapped onto existing SCCT categories, but some seem to be stand-alone approaches rather than fitting neatly into SCCT. Instead of force-fitting into SCCT, consider discussing how these strategies might complement or challenge the existing framework.

- We sincerely appreciate the insightful observations made by the reviewers regarding the relationship between our research findings and the SCCT framework. We believe that the reviewers' opinions might suggest that mapping the new strategies onto the existing framework of SCCT could potentially obscure its unique implications, especially in specific cultural contexts, thereby missing opportunities to challenge or expand the theory. We consider your opinion to be highly innovative and it has greatly inspired and enlightened us. However, after our in-depth discussion, we believe that making further changes to the core framework of this study in the fourth round of minor revision seems a little bit risky. Moreover, incorporating the newly discovered strategies into the SCCT framework is not entirely without basis. This is based on the previous research results of other scholars (as we mentioned in the previous response letter and the article), and secondly, our conclusion is reached through the consensus of multiple rounds of coding and discussions by the coders. We admit that the current framework is not perfect, and it is not immutable either, but if the framework is modified, it might require the coders to have a deeper discussion and potentially overturn and rewrite the main part of this article. Nevertheless, your suggestions have greatly inspired us. We will deeply reflect on your suggestions and hope to achieve further innovation in subsequent research. Thank you for your professional advice and we hope that you can accept and understand our explanation for this matter.

4. Condense the discussion of response strategies per crisis cluster to avoid redundancy.

- Thanks to the reviewer's suggestion. According to your suggestion, firstly, we reorganize the discourse of the discussion about applicable response strategies for different crisis clusters in section 4.4 by streamlining the expression without reducing the content, and consider the sentence changes to enhance readability. Secondly, we simplified and modified the redundant content in the conclusion paragraph of section 4.4, that is, the last paragraph of section 4.4. The revised contents are as follows:

“4.4 Applicable response strategies for different crisis clusters

SCCT emphasizes the adaptation of crisis response strategies to different situations. This study conducts a comparative analysis of the use of crisis response strategies in different situations. As Fig 2 shows, in the intentional cluster, the primary strategy of rebuild, including compensation, apology, commitment, and remedy, are most frequently employed. Chinese universities tend to use accommodative strategies when responsibility attribution is strong. Accountability and punishment strategies are also common, reflecting a similar accommodative approach. Endorsement, concern, and condolence are the most used secondary strategies.

For the accident cluster, the strategy of diminish, including excuse, transference, and separation are prevalent. These strategies are optimal in situations with moderate responsibility attribution. Additionally, explanation is a key primary strategy, and reminder and warning are common secondary strategies which are well-suited to crises in the accident cluster.

In the victim cluster, the primary strategy of deny, which includes attack the accuser, denial, and scapegoat, are widely used. Although the public might not hold the universities directly responsible for the crisis in this situation, restoring public trust depends on the universities’ response. Deny is effective in stabilizing public emotions with minimal cost in situations with weak responsibility attribution. Appeal, valued highly, gratitude, and statement are secondary strategies frequently employed. Notably, investigation and information disclosure strategies were applicable across all crisis situations.

Therefore, the results of this study prove that the actual situation of crisis response in Chinese universities is consistent with the concept of SCCT, that is, the degree of accommodation of applicable strategies increases with the degree of responsibility. The conclusion of “the rebuild strategies are the safest crisis response for the reason of rebuild strategies addressing victims well” is not universally applicable, because using overly accommodating strategies in the inappropriate situations makes the cost higher to the organization but may not see any increased yield in reputation protection. Therefore, the effectiveness of the response to the crisis is not due to the unlimited accommodative response strategy, but to the adaptation of the response strategy to the category of the crisis. The results of this study provide a summary of recommendations for the use of crisis response strategies in Chinese universities.”

5. Table 5 requires restructuring for clarity. Consider sentence variation to improve readability.

- Thanks to reviewer for your professional advice. Actually, during the first round of revision, Reviewer #1 deemed there to be too many tables in the manuscript and suggested removing some of them, while describing the contents of the tables in simple text without losing any details. Therefore, in the first round of revision, we have already removed Table 5 and summarized it in a simple way. We are not yet certain if a minor misunderstanding within the review system has led to a discrepancy in information. To address this, we have carefully examined the revised manuscript we submitted, ensuring that there is no Table 5 included. We have also checked the files in the submission system and confirmed that Table 5 is not present. However, we have modified the relevant content to improve readability by increasing sentence variability. See the answer in comment 4 for details. We remain grateful for your review of our manuscript, and we will make every effort to address any other revisable comments.

We sincerely thank you again for your meticulous review of our research and your valuable comments. Your feedback is of great importance to us and has prompted us to make corresponding revisions and improvements to the paper. We are confident that these enhancements have enhanced the quality and clarity of the paper. We sincerely hope that after careful revisions, the manuscript will meet your approval and gain the opportunity for publication. Thank you once again for your hard work and professional guidance.
