# Supplementary material for: Choosing the right strategies: An analysis of crisis response strategies in Chinese universities
Source: PLoS One. 2025 Apr 8;20(4):e0321777. doi: 10.1371/journal.pone.0321777 (PMC11991675; doi:10.1371/journal.pone.0321777)
Supplement: Attachment — Submitted filename: Response to Reviewers.docx [file pone.0321777.s003.docx]

**Dear Editor and Reviewers:**

We sincerely thank you for your time and effort in reviewing this manuscript. Your professional comments have greatly helped to improve this study. We have followed your suggestions and conscientiously revised and responded to each comment. Thank you again for your valuable suggestions, and we sincerely hope that the revision of this manuscript can get your approval.

**Regarding revisions required by the editor:**

1. Please ensure that your manuscript meets PLOS ONE's style requirements, including those for file naming.

- Thank you for the reminder. We checked the manuscript style, and we believe the style has already met PLOS ONE's style requirements.

2. In your Methods section, please include additional information about your dataset and ensure that you have included a statement specifying whether the collection and analysis method complied with the terms and conditions for the source of the data.

- We declare that the collection and analysis method complied with the terms and conditions for the source of the data.

3. All PLOS journals now require all data underlying the findings described in their manuscript to be freely available to other researchers, either 1. In a public repository, 2. Within the manuscript itself, or 3. Uploaded as supplementary information.

- After anonymizing the names of all the people involved in the crisis, we agreed that the data would be available. We will do our best to meet the requirements of the journal. We have already uploaded all the data as supplementary information.

4. We notice that your supplementary tables are included in the manuscript file. Please remove them and upload them with the file type 'Supporting Information'. Please ensure that each Supporting Information file has a legend listed in the manuscript after the references list.

- Thanks for the reminder. We are Sorry for our oversight. We will make corrections when the revised manuscript is submitted

**Regarding revisions required by the reviewer#1:**

1.Abstract/ it is important to clarify the methodology of the research.

- Thanks to Dr. Zakaria Elkhwesky for your professional comments. According to your suggestion, the methodology of the research have been added in the abstract.

2.Introduction/ the authors must indicate more details about the crisis types in China and also which crises are the focus of the current study. Gap and what makes this study different are not obvious in the introduction.

- Thanks for your professional comments. Details about the crisis types in China and crises which are the focus of the current study, also the distinction of this study have been added to the manuscript. Due to the large amount of content, it is not shown in this letter. Please review the revised manuscript. We hoped that the revised manuscript has been improved.

3.Literature review/ all universities mentioned in this part should by clarified by adding the country.

- Thanks for the reminder. The universities mentioned in the prat of literature review have been clarified by add the country.

4.Methodology/ why the authors select 2016.08 to 2023.08? justification needs to be clarified.

- Regarding the reason why we select 2016.08 to 2023.08 as the time range for case selection, we have added further explanations to the manuscript.

“In this study, representative crises on Chinese social media from 2016.08 to 2023.08 are selected as the research objects, since the notice of “Further Improving Response Capabilities to Government Affairs Crises in the Work of Openness of Government Affairs” became a landmark in China's crisis response, which issued in August 2016 by Chinese government to require clearly that “governments at all levels and their departments should attach great importance to the response to public opinion crisis in government affairs and implement the responsibility of response". The release of the notice standardized the public opinion crisis response and promoted the public opinion crisis response to become the normalization of the government and various institutions. Since then, the response to the public opinion crisis in Chinese universities has also begun to increase gradually. Therefore, this study selected crises between the time when the notice was released to the end time as the research objects of this study.”

More information about how the authors analyzed data are required.

- Thanks to Dr. Zakaria Elkhwesky for your professional comments. After consideration, we did find that the section on data analysis in the initial manuscript was short of description in detail. Therefore, we have made revision in the manuscript. We have redescribed the process of data analysis in the manuscript. The main additions are as follows, minor changes were also made to the rest, which are reflected in the manuscript.

“In this study, two researchers participated in coding work. One is a doctoral student in public opinion crisis, and the other is a staff engaged in the work related to public opinion crisis response. In the first step, the strategies proposed in SCCT and references mentioned above are sorted out as the original identified codes and themes. The Chinese university response documents are coded in sentences, and potential new strategies that do not fit the predetermined codes are recorded in new codes. Generating codes requires marking interesting features of the data in a systematic way. Then, all the codes are collected and sorted, the strategies that fit the definitions are classified into the predetermined themes, and any potential new themes that do not fit the predetermined themes are temporarily placed in the miscellaneous category which is added to account for any incongruous evidence. The researchers investigate whether the themes have "central organizing concepts”. Researchers generate new themes and then name and define the new themes based on the central concepts.

For more clarification, it is significant to verify the transferability, dependability and confirmability of data in qualitative thematic analysis. The concepts of transferability, dependability and confirmability were thoroughly examined and proved in this study. Transferability is determined by achieving reference adequacy by archiving the second half of the dataset until the first half is analyzed, and then comparing the data in the second half with the first half. Dependability is verified by peer debriefing. One researcher provides another with detailed notes showing how he came to his findings, and another researcher provides an external check to ensure that the observations and interpretations of the data are valid. Confirmability is achieved by researchers detailing their notes in a systematic way to illustrate the connections between their data and findings. Through the verification, the coding scheme is basically determined.

In the second step, according to the coding scheme, coders were asked to code all the response documents sentence by sentence, but the same strategy used repeatedly in the same crisis was still recorded as once in the final count. To further ensure the full understanding and consistency of the coding scheme between the two coders, 20% of the documents were conducted preliminary test on request. Before the comprehensive implementation of the coding, any inconsistencies in coding or new discovered response strategies are introduced to a third researcher for further discussion until a consensus was reached.”

Also, the two questions mentioned in the method section should be showed.

- Thank you for your suggestion. The two questions mentioned in the method section have already been showed in the revised manuscript.

More details about how the authors conduct the content analysis are essential. The following reference could be helpful; Salem, I. E., Elkhwesky, Z., & Ramkissoon, H. (2022). A content analysis for government’s and hotels’ response to COVID-19 pandemic in Egypt. Tourism and Hospitality Research, 22(1), 42-59.

- We read the article in detail. The presentation of the methodology in this article is outstanding. We have made a lot of modifications to the part of method in the manuscript after referring to this article. Please check whether there has been any improvement of the part of method in the revised manuscript. Thank you for your comments and your reference, which has been an important guide for us.

5.Findings and discussion/ Tables should be less and summarized in an easy way.

- Thank you for your suggestion. In the manuscript, first, we deleted Table 3, the text in the manuscript is sufficient to describe the content in this table. Second, we removed Table 4 to present it by Fig 2. which is more aesthetically pleasing and minimalist. Also, we deleted Table 5 and summarized it in a simple way. Finally, we found that the contents of 4.4 and 4.5 can be combined, and we have made some adjustments to the structure of the conclusions of these two parts, hoping that the revised manuscript will be better.

6.Conclusion/ this part is very poor. So, it is important to divide it into two subtitles: theoretical contribution and practical implications.

- Thank you for your valuable advice. We read the conclusion part of the reference “*Salem, I. E., Elkhwesky, Z., & Ramkissoon, H. (2022). A content analysis for government’s and hotels’ response to COVID-19 pandemic in Egypt. Tourism and Hospitality Research, 22(1), 42-59”*in detail and studied the conclusion structure of the reference. We have rewritten the conclusion by divide it into two subtitles: theoretical implications and practical implications. Due to the large amount of content, it is not shown in this letter. Please review the revised manuscript. We hoped that the revised manuscript has been improved.

7.The English language should be revised by a native speaker.

- Thanks to Dr. Zakaria Elkhwesky for your advice. We will review the language of this article and seek help from our peers.

**Regarding revisions required by the reviewer#2:**

1. More LR needs to be added.

- Thanks for your professional comments. The manuscript has been extensively revised and the number of references has increased from 39 to 58.

2.Details of the methodology need to be provided.

- Thank you for your valuable advice. We did find that the section on data analysis in the initial manuscript was short of description in detail. Therefore, we have made revision in the manuscript. We redescribe the process in qualitative thematic analysis of RQ1. The description of the content analysis of RQ2 is also supplemented. Due to the large amount of content, it is not shown in this letter. Please review the revised manuscript. We hoped that the revised manuscript has been improved.

3.The basis of selection and choices in crises needs to be elaborated.

- Thank you for your suggestion. In this study, the basis of selection and choices in crises are constituted by 3 reasons as we mentioned in the part of data collection of the manuscript. First, the crisis has been on the list of hot topics on Sina Weibo, which proves that the crisis is with media attention and public discussion. Second, the crisis must be in the case-base of the National University Online Public Opinion Monitoring Center, this case-base is a collection of all the crises with topic sensitivity and event impact. Third, the public discussion of the crises which we selected in this study decreased significantly and faded within a week and no secondary public opinion crisis occurred after the issuance of the response document. We considered the response documents of Chinese universities in these crises to be initially effective in this study. In future studies, we will further improve the research by empirically analyzing the effectiveness of responses through public reaction.

4.How study can be fruitful to policymakers needs to be added.

- Thank you for your suggestion. Regarding how study can be fruitful to policymakers, we have rewritten the part of conclusion. Especially in the part of practical implication, we added some content, hoping to improve the manuscript.

“This study has useful implications for the practitioners and policymakers of crisis response in Chinese universities. The results of this study not only provide strategies options for the practitioners and policymakers of crisis response in Chinese universities, but more importantly, according to the demand of SCCT, a link between crisis situations and crisis response strategies should be established.”

5.Check the referencing style.

- Thanks for reminding. We have re-checked the referencing style. We are Sorry for our oversight.

Thanks again to all the editors and reviewers for your professional suggestions on the revision of this manuscript. We hope that our revision of the manuscript can get your approval. If there are any suggestions still worth making, we would appreciate your reminder and your patience. We'll do everything we can to make the manuscript the best it can be.
