# Supplementary material for: Choosing the right strategies: An analysis of crisis response strategies in Chinese universities
Source: PLoS One. 2025 Apr 8;20(4):e0321777. doi: 10.1371/journal.pone.0321777 (PMC11991675; doi:10.1371/journal.pone.0321777)
Supplement: Attachment — Submitted filename: Response_to_Reviewers_auresp_2.docx [file pone.0321777.s004.docx]

**Dear Editor and Reviewer:**

We sincerely thank you for the time and effort you spent providing professional comments on our articles. As you are concerned, there are still several issues that need to be addressed. We have carefully reconsidered these problems and revised them one by one according to your suggestions. We sincerely hope that the revision of this manuscript can get your approval.

**Regarding revisions required by the editor:**

1.Please review your reference list to ensure that it is complete and correct. If you have cited papers that have been retracted, please include the rationale for doing so in the manuscript text, or remove these references and replace them with relevant current references. Any changes to the reference list should be mentioned in the rebuttal letter that accompanies your revised manuscript. If you need to cite a retracted article, indicate the article’s retracted status in the References list and also include a citation and full reference for the retraction notice.

- Thanks to the editor for the reminder. We re-examined the references individually to confirm that they had not been retracted. In addition, we have added several references during the revision of the manuscript. The added references are listed below：

[1] Leibold J. Blogging alone: China, the internet, and the democratic illusion? The Journal of Asian Studies. 2011;70 (4):1023–1041.

[2] Weng SH. Participation-response model: An analytical framework of the government response to internet political participation for decision-making. Journal of Public Administration. 2014;7(05):109-130+191.

[3] Sun LM. The rise of civil rights consciousness: A largely internet-based observation. Journal of the Central Institute of Socialism. 2010;(03):99-103.

[55] Vaismoradi M, Turunen H, Bondas T. Content analysis and thematic analysis: Implications for conducting a qualitative descriptive study. Nursing and Health Science. 2013;15,398-405.

[56] Hayes AF, Krippendorff K. Answering the call for a standard reliability measure for coding data. Communication Methods and Measures. 2007; 1:77-89.

Also, we have double-checked the references format and carefully revised it to ensure that they have met the journal's requirements for the Vancouver style.

**Regarding revisions required by the reviewer #2:**

1.The revisions should be highlighted and put as the main text, not after the original manuscript. In text, citations should be given rather than the numbers.

- Thank to the reviewer for the reminder. For the main parts of the revision, we have highlighted it in green font in the main text. We have carefully considered and revised the article according to your valuable suggestions. In addition, citations have been modified as required by the journal and your suggestion, also we highlighted the changes to citation in the article for your review. We are sorry for our carelessness and thank you again for your reminder.

2.The revised introductions need to be rephrased. It should be in the form of academic writing, not as a normal conversation.

- Thanks for your professional comments. We have reconsidered and rewritten the part of introduction. The revised introduction is more academic than introductory. We deleted the simple introduction of the type of public opinion crisis on the internet in China, and strengthened the explanation of why the internet has become the most important channel for Chinese citizens to express their opinions and demands:

“Unlike some Western developed countries, they have many offline institutionalized channels for citizen participation such as public hearings and citizens’ assemblies in addition to online participation. In China, there is still a contradiction between the narrow channels for citizen participation and the enthusiasm of modern citizens for political participation. Therefore, the convenience of social media has made it the most convenient way for Chinese citizens to express opinions and seek solutions to problems from the government. The expression of opinions and demands through online participation has become the most active part of Chinese citizens in the public sphere of the internet.”

Your valuable suggestion helps to improve the quality of our article, and we hope the revision can get your approval.

3.Elaborate more on the technique used for the content analysis.

- Thanks to reviewer for your professional advice. After re-examination, we did find that the description of the content analysis section was not detailed enough, and we omitted many details during the writing process, which were not shown in the article. We have re-described this part in detail, hoping to explain the process of content analysis clearly. The main modifications are as follows:

“Content analysis is one of the most important research techniques in social sciences to analyze data with a specific context. Thus, when using content analysis, the researchers should have a broader understanding of the context. Content analysis provides a systematic and objective means to make valid inferences from verbal, visual, or written data to describe and quantify specific phenomena in a conceptual form. There are three main steps in content analysis: (a)obtaining the sense of the whole data, selecting the unit of analysis, (b)creating categories and open coding, (c)reporting the analysis process and the results through conceptual system.

In this section, taking a case as a unit, the two coders coded independently to divide Chinese universities crises into three clusters after fully understanding the relevant concepts of crisis classification based on SCCT and the research requirements of classification category. The victim cluster, the accident cluster and the intentional cluster are the three first-level codes of this coding process. First, 20 cases were randomly selected for independent coding. After coding, consistency judgment is carried out. A reliability of 0.863 is achieved using Krippendorf’s alpha, which is higher than the standard of 0.7 and shows that two coders have a high degree of agreement in understanding data, context, and coding concepts in the content analysis. Also, the sample size of preliminary test meets the requirement of 10%-20% of the total samples. All remaining disagreements were completely discussed prior to the next implementation of coding until a consensus was reached. In the second round, 50 cases were randomly selected from the remaining cases for independent coding. The coding with inconsistent results were discussed prior to the next implementation of coding until a consensus was reached. In the third round, the remaining 77 cases were coded independently, and the coding with inconsistent results were discussed until a consensus was reached. The discussion after each round of coding makes the next round of coding less disagreement, and the conceptual system of crisis classification of Chinese universities are basically formed, which provides clues for Chinese universities to classify the crisis clusters quickly and reliably. After three rounds of coding, 147 cases were finally classified by the degree of responsibility.”

As Vaismoradi M, Turunen H and Bondas T (2013) proposed, “the creativity of the researcher for presenting the result in terms of conceptual system is encouraged in the content analysis”. Actually, in the coding process of content analysis, the researchers gradually formed a conceptual framework for crisis classification of Chinese universities, and finally divided 147 cases into three clusters according to this conceptual system. The conceptual system further refines the brief definition of crisis cluster classification of Chinese universities based on theoretical framework of SCCT. The specific content has been added to “4.3 Crisis cluster in Chinese universities”. The summary is as follows for the your review：

“According to the degree of responsibility attribution, the crises in Chinese universities can be divided into three clusters: (1) the victim cluster has very weak attributions of crisis responsibility and mild reputational threat. In the victim cluster, the university is also a victim of the crisis. These crises include the following situation: rumor incidents, natural disasters and unexpected accidents which damaged the reputation of universities; (2) the accidental cluster has minimal attributions of crisis responsibility and moderate reputational threat. In the accidental cluster, the university actions leading to the crisis are unintentional without causing substantial harm or the university is blamed for the leadership responsibility of individual behavior that have a huge impact. (3) the intentional cluster has very strong attributions of crisis responsibility and severe reputational threat. In the intentional cluster, the university knowingly placed people at risk, took inappropriate actions or violated a law or regulation. These crises include the following situation: improper management of laboratory or security resulting in death or injury, the universities violate the rules of academic fairness and examination fairness which makes a huge impact and even causes damage to the credibility of the entire education system.”

4.Refer to other seminal work on content and thematic analysis.
e.g.,

Liñán, F., & Fayolle, A. (2015). A systematic literature review on entrepreneurial intentions: citation, thematic analyses, and research agenda. International entrepreneurship and management journal, 11, 907-933.

Kim, H., & So, K. K. F. (2022). Two decades of customer experience research in hospitality and tourism: A bibliometric analysis and thematic content analysis. International Journal of Hospitality Management, 100, 103082.

Vaismoradi, M., Turunen, H., & Bondas, T. (2013). Content analysis and thematic analysis: Implications for conducting a qualitative descriptive study. Nursing & health sciences, 15(3), 398-405.

Thompson, J. (2022). A guide to abductive thematic analysis.

- Thanks to reviewer for your professional support and guidance. We spent a lot of time reading these references recommended by reviewer in detail and got a deeper understanding of content analysis and thematic analysis. These articles provide a detailed introduction to content analysis and thematic analysis. Based on these seminal literatures on the two methods, first, we re-examined the thematic analysis process in our article to ensure its accuracy and standardization. Second, we have also re-examined the content analysis process in our article and supplementary descriptions of the omitted details are provided, as you suggested. The references you recommended will also help us maintain academic rigor and normativity in implementing content analysis and thematic analysis in our research in the future. Thank you again for your professional advice.

5.Recheck referencing style. It is better if APA style is used as per the guidelines.

- Thank you for your suggestion. We reviewed again the reference requirements on the journal page. According to the journal requirements, the Vancouver style should be use: *“PLOS uses the reference style outlined by the International Committee of Medical Journal Editors (ICMJE), also referred to as the ‘Vancouver’ style”.* Therefore, we have not changed the style in this revision. However, in the process of re-checking, we confirmed that there were many formatting errors, and we have revised them one by one. Thanks again for the reviewer's reminder.

Finally, according to the journal requirement that the manuscript should be presented in an intelligible fashion and written in standard English, we have asked for help from professional language polishing service, hoping to improve the language of this article. The certificate is as follows:

Thanks again to editor and reviewer for your professional suggestions on the revision of this manuscript. Your valuable comments have greatly improved this manuscript. We have carefully revised the manuscript according to your suggestions and hope that our revision can get your approval. If there are any suggestions still worth making, we would appreciate your reminder and your patience. We promise that we will do everything we can to make the manuscript the best it can be.
