# Supplementary material for: Choosing the right strategies: An analysis of crisis response strategies in Chinese universities
Source: PLoS One. 2025 Apr 8;20(4):e0321777. doi: 10.1371/journal.pone.0321777 (PMC11991675; doi:10.1371/journal.pone.0321777)
Supplement: Attachment — Submitted filename: Response_to_Reviewers_auresp_3.docx [file pone.0321777.s005.docx]

**Dear Editor and Reviewers:**

We are deeply grateful and sincerely appreciate your generous investment of valuable time and energy in providing professional and in-depth review comments on our article. Even after two rounds of meticulous revisions, you maintained a rigorous attitude, carefully reviewed the manuscript again, and put forward detailed and invaluable suggestions. We hold your professionalism in the highest esteem and have learned invaluable academic attitudes from you. Based on your expert insights, we have once again reviewed the manuscript and made targeted improvements to its shortcomings. We eagerly anticipate and sincerely hope that the revised manuscript will meet with your approval and endorsement.

Furthermore, as we usher in the new year, we would like to take this opportunity to extend our warmest New Year wishes to the editor and reviewers of this article. Wish you all the best in 2025!

**Regarding revisions required by the editor:**

1.Please review your reference list to ensure that it is complete and correct. If you have cited papers that have been retracted, please include the rationale for doing so in the manuscript text, or remove these references and replace them with relevant current references. Any changes to the reference list should be mentioned in the rebuttal letter that accompanies your revised manuscript. If you need to cite a retracted article, indicate the article’s retracted status in the References list and also include a citation and full reference for the retraction notice.

- Thanks to the editor for the reminder. We re-examined the references individually to confirm that they had not been retracted. In addition, we have deleted several references during the revision of the manuscript. The deleted references are listed below:

[3]	Leibold J. Blogging alone: China, the internet, and the democratic illusion? The Journal of Asian Studies. 2011;70 (4):1023–1041.

[4]	Weng SH. Participation-response model: An analytical framework of the government response to internet political participation for decision-making. Journal of Public Administration. 2014;7(05):109-130+191.

[5]	Sun LM. The rise of civil rights consciousness: A largely internet-based observation. Journal of the Central Institute of Socialism. 2010;(03):99-103.

**Regarding revisions required by the reviewer #2:**

1.The abstract is informative but could better emphasize the study's practical implications and theoretical contributions. The identification of new strategies should be explicitly highlighted as an extension of SCCT.

- Firstly, we would like to express our sincere gratitude to the reviewer for acknowledging and recognizing our work. Secondly, based on your comments, we have revised the abstract to emphasize the theoretical contributions and practical significance of the research, as well as to highlight that the new strategy is an extension of SCCT. The main modifications of abstract are as follows:

“The findings revealed that Chinese universities have adopted 17 new strategies beyond the strategies proposed by SCCT, highlighting a discrepancy between the theoretical ground of the theory and the application of strategies in real-life crisis response of Chinese universities. Furthermore, the results revealed the applicable strategies for different crisis clusters, which contributed to construct the framework of crisis response strategies for Chinese universities in different situations. This study expanded SCCT theoretically by enriching the crisis response strategies while practically improving the applicability of SCCT in Chinese universities. The findings provided guiding significance to the crisis response for Chinese universities in different crisis situations, also its innovative strategies provided empirical evidence for other organizations in different cultural contexts in crisis response.”

2.The introduction successfully sets the context but includes some verbose sections. The discussion of the public’s enthusiasm for political participation can be streamlined to maintain focus on crisis response.

- Thanks to reviewer for your acknowledgment and suggestions. We have removed the lengthy discussion on public enthusiasm for political participation and emphasized the focus on crisis response. Your professional advice has made the introduction more concise and clearer. The main modifications of introduction are as follows:

Firstly, we deleted the verbose sections of“the internet has become the most important channel for Chinese citizens to express their opinions and demands. Unlike some Western developed countries, they have many offline institutionalized channels for citizen participation such as public hearings and citizens’ assemblies in addition to online participation. In China, there is still a contradiction between the narrow channels for citizen participation and the enthusiasm of modern citizens for political participation. Therefore, the convenience of social media has made it the most convenient way for Chinese citizens to express opinions and seek solutions to problems from the government. The expression of opinions and demands through online participation has become the most active part of Chinese citizens in the public sphere of the internet”.

Secondly, we have redescribed the first half of the introduction. The revised introduction maintain focus on crisis response as you suggested:

“With the development of social media in China, the Chinese public has increasingly gained the right to publish information and opinions. Public's awareness of political participation has been continuously enhanced, and the active degree of public speech and enthusiasm for political participation have reached an unprecedented height. Public opinion crises on social media have frequently occurred in China. In recent years, numerous crises in the fields of public health, food safety, medical malpractice, government management, product quality, security incidents, corporate image have aroused widespread public concern and discussion. The occurrence of public opinion crisis makes the relevant responsible parties under immense pressure to crises response. When public opinion crises occur, if the government, institutions, and enterprises fail to respond reasonably, it will cause great harm to their own image, lead to public opinion crises, and reduce credibility. Obviously, public opinion crisis response has become an important research topic at present.

Academics have shown great interest in research of these public opinion crises in China, and the relevant researches mainly focus on three points. First, researchers deeply discussed the interaction between government crisis management and public opinion, and analyzed the influence of public opinion on government management strategies and how the government effectively responds to these public opinions. Second, some researchers are committed to revealing the evolution law of public opinion, providing valuable insights for formulating effective response strategies through big data and computer models. Third, many researchers have conducted research on how enterprises and brands respond to negative public opinions on Chinese social media during crises. The research on public opinion crisis and crisis response is very important, which not only has profound theoretical value, but also shows its indispensable importance in reality.”

3.The gap in literature could be more concisely articulated to underline the necessity of this research.

- Thanks to reviewer for your professional advice. According to your advice, we redescribed the gap of literature and underlined the necessity of this research.

Firstly, we deleted the verbose sections of “There are quite few studies taking Chinese universities as the research object. The research on response strategies in China mainly focuses on government administrative departments, enterprises, and brands. Also, most of the research on crisis response in Chinese universities is from a macro perspective and there are short of empirical research.”

Secondly, it was concisely expressed as “However, despite the prevalence of crises in Chinese universities, there is limited research on how to respond. In particular, empirical studies on crisis response in Chinese universities, are almost nonexistent”.

Also, we further underlined the necessity of this research that “the findings of this study provide a comprehensive and definitive answer to the question that has yet to be addressed in current research: what strategies are used in the public opinion crisis response in Chinese universities and what strategies are applicable to different types of crises”.

4.The SCCT framework is adequately explained, but the integration of new strategies into the framework appears to be forced.

- Regarding your concern that the integration of the new strategies into this framework appears to be forced, we understand your perspective. Please allow us to explain in detail. In fact, in many studies on crisis response strategies based on the Situational Crisis Communication Theory (SCCT), researchers often incorporate new strategies into the SCCT framework. Take several articles mentioned in the literature review section of this study as examples. In fact, the integration of new strategies into the SCCT framework in this study is based on the following research findings.

Castonguay and Lowes (2022) [39] conducted a thematic analysis of the National Football League's (NFL) response to the concussion crisis and found that the NFL employed some new strategies, among which they integrated the strategy of “organizational change” into the “rebuild” strategy within the SCCT framework.

Kim and Liu (2012) [37] applied SCCT to investigate how 13 corporate and government organizations responded to the first phase of the 2009 flu pandemic through a quantitative content analysis, and expanded SCCT's response strategy options of “enhancing” and “transferring” and integrates them to a new theme called "other strategy”.

Lai and Tang (2018) [38] discovered six new strategies not mentioned by SCCT by analyzing 64 cases of Chinese public opinion crisis. They integrated the new strategies of “gratitude”, “accountability”, “punishment”, and “explanation” into the “primary strategies” and incorporated the new strategies of “information disclosure” and “attitude indication” into the “secondary strategies” of the SCCT framework.

Liu (2010) [35] applied SCCT to identify how organizations and individuals can effectively respond to racially charged crises through a content analysis of 104 response documents and 144 newspaper articles. He expanded SCCT by identifying several strategies that organizations and individuals used to respond to racially charged crises that were currently not included in the theory. In his study, the new strategy of “ignore” was integrated into the “deny” strategy within the SCCT framework, the new strategy of “separation” was integrated into the “diminish” strategy, and the new strategy of “transcendence” was integrated into the “rebuild” strategy. Additionally, he introduced a new theme called “reinforce” and incorporated a new strategy called “endorsement” into it (as shown in the figure below).

5.Coder bias, or cultural context influencing the interpretation of data, is not discussed adequately. The rationale for excluding third-party narratives is valid but should be elaborated with respect to the exclusion of stakeholder perceptions.

- Thank you for your valuable suggestion. As pointed out by reviewer, coder bias, which refers to the influence of cultural context on data interpretation, does indeed exist. While this manuscript mentions it, the discussion is not sufficient. Therefore, we have made some revisions to address this issue:

“due to the influence of cultural context on the interpretation of data, it is worth noting that although the explanations and the example sentence for each strategy in this study were translated into English, researchers actually used Chinese during the analysis, because analysis in the original language of the document can fully reveal the use of strategies in the particular context, so as to ensure the accuracy and effectiveness of the analysis results.”

Besides, regarding your comment, "the rationale for excluding third-party narratives is valid but should be elaborated with respect to the exclusion of stakeholder perceptions," we have made the following modifications accordingly.

“As the purpose of this research is to determine how the Chinese universities communicated about crises in their external communications, news articles or interviews of school staff were excluded to avoid the subjectivity of a third party in the reporting and framing of the universities’ responses. All documentary evidence is officially released by universities. Meanwhile, due to stakeholders perceptions may be influenced by their own interests, and potential emotional factors and biases towards the organization, in order to focus on the response strategies of Chinese universities and maintain the objectivity of the research, the influence of stakeholders perceptions are also excluded in this study.”

6.The findings are repetitive in parts. The description of strategies used in each crisis cluster could be condensed without losing essential details.

- Thank you for your suggestion. Upon your reminder, we have reviewed the findings section of the article again and found that there are indeed repetitive parts, especially in section 4.4. Therefore, we have carefully revised this section, and the specific revisions are as follows:

Firstly, we deleted the redundant parts: “Based on the research above, this study provides a summary of recommendations for the use of crisis response strategies in Chinese universities. For the crises of victim cluster, it is applicable to use the strategies of attack the accuser, denial, scapegoat, endorsement, appeal, valued highly gratitude and statement. For the crises of accident cluster, the strategies of excuse, transference, separation, explanation, reminder, warning, concern, condolence and gratitude are the reasonable choice in response. For the crises of intentional cluster, the strategies of compensation, apology, commitment, remedy, accountability, punishment, endorsement, concern and condolence are suitable in this situation. The study also reveals that the strategies of investigation and information disclosure can be applied to any situation in the public opinion crisis response in Chinese universities.”

Secondly, we have condensed the description of the strategies used in each crisis cluster without losing essential details. We compress the length by using more concise expressions like “Therefore, it is applicable to use these strategies for the crises of Chinese universities in the victim cluster” while preserving the original meaning. Due to the extensive length of the modifications, specific changes have been marked in different colored fonts in the manuscript. We kindly request the reviewer to review the manuscript and hope that the revised findings will meet your satisfaction.

7.The discussion section does not sufficiently explore the implications of the findings for global applications of SCCT.

- Thank you to the reviewer for your professional and meticulous comments. Based on your suggestions, we have supplemented information regarding the impact of our research findings on the global application of SCCT. Due to the excessive length, only the added contents are shown in this response letter. The specific additions are as follows:

“This study not only deepens the understanding of crisis response strategies in Chinese universities but also makes significant contributions to the application and development of SCCT globally. This study enriches and expands the theoretical framework of SCCT. Through empirical research in the specific context of Chinese universities, it not only validates the basic framework of SCCT, comprehensively explores the strategies used by Chinese universities in crisis response but also further refines the classification of crises and the strategies that should be adopted for different crisis types.

This study facilitates a more precise application and adaptation of SCCT in different cultural and contexts worldwide. As China's influence on the global stage continues to grow, research findings on crisis response in Chinese universities will attract more international attention. Conducting research on crisis response by applying SCCT within the Chinese context. not only helps enhance the international applicability of SCCT but may also inspire more countries and regions to engage in research and practice related to this theory, thereby promoting its widespread application and recognition globally.

The research findings of this study also contribute to other organizations or academic research worldwide. This study enhances the practicality and operability of SCCT, and its findings contribute to enabling universities, governments, and enterprises globally to develop and implement communication strategies more rapidly and effectively when facing crises.

The research results of this study offer a foundation for cross-cultural comparisons. The research on crisis response strategies in Chinese universities provides a valuable case for cross-cultural comparisons within the context of SCCT. By comparing crisis management practices in Chinese universities with those in Western or other regional universities, it can reveal the similarities and differences in crisis communication strategies across different cultural and social backgrounds, thereby promoting the exchange and integration of crisis management knowledge globally.”

8.The manuscript has undergone professional editing; minor grammatical issues and awkward phrasing persist, such as "unnecessary situations" (page 23) and "radically increasing" (page 14). Sentence structures could be varied to improve readability.

- Thank you for the reviewer's reminders. Your professionalism and seriousness towards academics are admirable and worthy of our learning. We have made corrections to some grammatical issues and awkward phrasing. Specifically, we have changed "unnecessary situations" to "inappropriate situations" and "radically increasing" to "greatly increasing". "Radically" is an adverb whose basic meaning is "fundamentally, thoroughly, or extremely." It is suitable for emphasizing the depth and thoroughness of a certain action, change, or viewpoint. While the expression "unnecessary" is grammatically correct, it may lack precision semantically or be prone to causing ambiguity. In this context, "unnecessary" is usually used to describe something or an action as unneeded or redundant, and changing it to "inappropriate" aligns more closely with the intended meaning of this text. Thank you again for your reminders. Your professional and conscientious attitude is worthy of our learning.

9. The conclusion reiterates key findings. It should also emphasize their significance for theory and practice. Additionally, it should provide scope for future research.

- Thank you for your professional comments. Firstly, in the conclusion section, in addition to reiterating the key findings, we have divided the significance of the research into two parts for discussion: theoretical implications and practical implications. Due to the extensive content, we have not included it in this response letter. We kindly request the reviewer to review the manuscript for the additions. Secondly, this study offers three potential research directions for the future:

“Firstly, SCCT proposes that crisis history and prior relational reputation have both direct and indirect effect on the reputational threat posed by the crisis. The two factors were not considered in this study, but it is valuable to further investigate in future research. Secondly future research should explore the combinations of strategies employed in different crises. The findings of this study indicate that Chinese universities employed 4.01 response strategies on average in each crisis, suggesting that Chinese universities typically use multiple strategies in crisis response. Future research should further explore whether there are fixed combinations of these strategies. Thirdly, in future studies, the attributions of crisis responsibility can be determined by news media and public opinion by quantitative methods to increase the credibility of the findings in the future.”

**Regarding revisions required by the reviewer #3:**

1. ROWS 23-25, needs more clarification by adding some values that support the topic. Also, you should highlight the most adopted strategies by universities (maximum no. of response).

- We sincerely appreciate the effort and time you have devoted to providing revision suggestions for our research. Your professional insights are extremely valuable for us. We will endeavor to make revisions and provide explanations for each of your suggestions.

Based on the comment 4.ROW 302 (shown in S1 Table) and comment 5. Table 5, we speculate that your review comments were based on the initial version of the manuscript. Therefore, the abstract you are seeing may be the initial version.

Based on your feedback, ROWS 23-25 may be the final sentence in the abstract. Adding some values that support the theme and emphasizing that the strategy most commonly used by Chinese universities indeed highlights the research findings and its significant value. However, after careful consideration and detailed discussion, we ultimately decided not to include these additions. Please allow us to explain to you as follows:

Firstly, the research focus of this study is divided into two points. The first is to explore the strategies employed by Chinese universities in crisis response by identifying, analyzing, naming, and summarizing those newly discovered strategies through thematic analysis. The second is to classify the crises of Chinese universities through content analysis and discuss which strategies were used in different situations. Therefore, the most significant value that can support the topic is that this study has identified 24 strategies used by Chinese universities in crisis response, of which 17 are strategies beyond those of SCCT (Situational Crisis Communication Theory). This value has already been included in the abstract. In addition, the results of this study show that the most adopted strategies by Chinese universities in crisis response are valued highly (68), punishment (66), information disclosure (63), commitment (63) and investigation (59). The differences between these values are not significant, so merely mentioning the most frequently used strategy does not seem to have much meaning. But if all of them are mentioned in the abstract, it would appear rather lengthy. Moreover, this content is not the most important topic of this study. The abstract of this study after revisions, besides introducing the research methods, data, and focuses, also emphasizes the practical significance and theoretical contributions of the research. Adding values beyond the research focus would make it slightly more complicated.

Therefore, in this revision, we have not included these values in the abstract. Please allow us to explain this to you in detail, hoping that our explanation will provide you with a clear understanding. If you still have suggestions for further revision of the abstract, we will make every effort to address them. Once again, Thank you again for your professional comments.

2. ROWS 31-36, needs to be reformulated.

- Thank you for your advice. Based on your professional advice, we have revisited the content from ROWS 31 to 36 and identified issues with unclear expression and non-standard terminology. We have meticulously revised it to ensure more precise language and adherence to academic norms. The revised content is as follows, and we hope to gain your approval.

“In recent years, numerous crises in the fields of public health, food safety, medical malpractice, government management, product quality, security incidents, corporate image have aroused widespread public concern and discussion. The occurrence of public opinion crisis makes the relevant responsible parties under immense pressure in crises response. When public opinion crises arise, if governments, institutions, and enterprises fail to respond reasonably, it will cause severe damage to their own image, trigger further public opinion crises, and undermine their credibility.”

3. Table 1, case No. 9, Add an explanation to this case that shows its strength.

- Thank you for the reviewer's reminder. Your review of this paper was very thorough. Case 9 is indeed an important and influential representative case, and based on your suggestion, we have enhanced its description in terms of intensity. However, due to the description in the table is not appropriate to be too long, we have redescribed it as "A well-known actor committed academic fraud during his doctoral and postdoctoral studies which damaged the credibility of Chinese education system seriously.” We hope that this modification will bring about improvements.

4. ROW 302, (shown in S1 Table) …It is preferable to refer to the section and page.

- Thank you for your suggestion. Since S1 Table is supplementary information and is not displayed in the main text, mentioning its section may help readers locate it more quickly. Therefore, based on your suggestion, we have added the section for it, that “Real-life examples of each strategy are shown in S1 Table in the section of supporting information”. However, since the supplementary information will be a hyperlink and it may not have a page number, we have not added the page. We hope that our modifications have met your suggestion, and the explanation have clearly conveyed to you.

5. Table 5, rearrange the table.

- Thanks to reviewer for your professional advice. Actually, during the first round of revision, Reviewer #1 deemed there to be too many tables in the manuscript and suggested removing some of them, while describing the contents of the tables in simple text without losing any details. Therefore, in the first round of revision, we have already removed Table 5 and summarized it in a simple way. We suspect that due to a slight misunderstanding in the review system, you may have seen the initial version of the manuscript. We remain grateful for your valuable suggestions and will make every effort to revise all possible sections based on your professional comments.

6. ROW 399, numeric values should be contained.

- Thank you for your comments. Based on the comment 4.ROW 302 (shown in S1 Table) and comment 5. Table 5, we speculate that your review comments were based on the initial version of the manuscript. Therefore, according to the initial version of the manuscript, ROW 399 should be "Conclusion." We have double-checked the format to ensure that it includes the numeric value“5. Conclusion”. Thank you for your reminder.

We sincerely thank you again for your meticulous review of our research and your valuable comments. Your feedback is of great importance to us and has prompted us to make corresponding revisions and improvements to the paper. We are confident that these enhancements will enhance the quality and clarity of the paper. We sincerely hope that after careful revisions, the manuscript will meet your approval and reach the standard for publication. We look forward to having more opportunities for academic exchanges with you in the future. Thank you once again for your hard work and professional guidance.

Wishing Editor Dr. Ahmed Meri, anonymous Reviewer#2 and Reviewer Dr. Zahraa Talib a prosperous and joyful 2025, filled with endless opportunities, achievements, and happiness. May this year bring you health, wealth, and success in all your endeavors.
